# Adverse Mental Health Sequelae of COVID-19 Pandemic in the Pregnant Population and Useful Implications for Clinical Practice

**DOI:** 10.3390/jcm11082072

**Published:** 2022-04-07

**Authors:** Dariusz Wojciech Mazurkiewicz, Jolanta Strzelecka, Dorota Izabela Piechocka

**Affiliations:** 1 St. Mark’s Place Institute for Mental Health, 57 St. Mark’s Place, New York, NY 10003, USA; 2 Department of Neurology and Pediatrics, Medical University of Warsaw, Żwirki and Wigury 61 Street, 02-091 Warsaw, Poland; jolanta.strzelecka@wum.edu.pl; 3 Department of Gynecology and Practical Obstetrics, Medical University of Bialystok, Szpitalna 37 Street, 15-295 Bialystok, Poland; dorota.piechocka@umb.edu.pl

**Keywords:** COVID-19, psychological distress, anxiety, depression, pregnancy, disaster, telemedicine, theory–practice gap, stress, trauma

## Abstract

The COVID-19 pandemic has increased risk of disturbances in the functioning of everyday life, directly or indirectly has influenced the risk of mental disorders in the most vulnerable populations, including pregnant women. The aim of this study was to analyze adverse mental health effects in the pregnant population during the COVID-19 pandemic, investigate risk factors for adverse mental health outcomes, identify protective factors, and create practical implications for clinical practice, bearing in mind the need to improve perinatal mental healthcare during such pandemics. Qualitative research was conducted in the electronic databases PubMed and Web of Sciences for the keywords COVID-19, pregnancy, depression, anxiety, and telemedicine for relevant critical articles (*n* = 3280) published from 2020 until October 2021, outlining the outcomes of control studies, meta-analysis, cross-sectional studies, face-to-face evaluation survey studies, remotely administered survey studies, and observational studies regarding the main topic; all were evaluated. Mental health problems among pregnant women linked to the COVID-19 pandemic, in most cases, show symptoms of depression, anxiety, insomnia, and PTSD and may cause adverse outcomes in pregnancy and fetus and newborn development, even at later stages of life. Therefore, useful implications for clinical practice for improving the adverse mental health outcomes of pregnant women associated with the COVID-19 pandemic are highly desirable. Our research findings support and advocate the need to modify the scope of healthcare provider practice in the event of a disaster, including the COVID-19 pandemic, and may be implemented and adopted by healthcare providers as useful implications for clinical practice.

## 1. Introduction

The COVID-19 pandemic, as a public health emergency of international concern, constitutes a challenge to psychological resilience [1], heavily impacting global mental health [2] by causing acute respiratory coronavirus 2 (SARS-CoV-2) [3], with a rapid increase in the number of cases and deaths since its first identification in Wuhan, China, in December 2019 [4]. The vulnerable population of women who became mothers during the COVID-19 emergency appear to be at high risk for developing mental health problems [5]. Respiratory fluid droplets containing the SARS-CoV-2 virus may spread from person to person—in some cases, through contact with an infected surface when the person touches their eyes, nose, or mouth—and the average SARS-CoV-2 incubation period is 3–7 days [6]. The symptoms of COVID-19 thus may or may not be observed, and this disease can be confirmed by laboratory test results. Pregnant women may suffer from asymptomatic COVID-19 and an increased leukocyte count, raised lymphopenia, and higher C-reactive protein (CRP) levels, requiring extracorporeal membranous oxygenation (ECMO) and invasive ventilation more than nonpregnant women do [7]. The risk factors of severe COVID-19 in pregnancy are directly linked to preexisting diabetes, chronic hypertension, pre-eclampsia, and high body mass index (BMI); in the long term, outcomes such as higher risk of preterm birth (PTB) and/or pre-eclampsia may occur in pregnant women who have suffered from COVID-19 [8]. The risk of vertical SARS-CoV-2 infection in pregnancy, and its related fetal growth restriction, miscarriage, and preterm births are still quite unclear and under deliberation [9], and neonates born to mothers infected with SARS-CoV-2 have an overall favorable prognosis [6]. However, there is a clear link between poor mental health in pregnant women and pregnancy complications [10]. Mental health problems have been observed and reported to increase in the pregnant population in general, including an increase in the incidence of depression, antenatal, postpartum depression, and anxiety, all associated with adverse effects on intrauterine growth, birth weight, prematurity, behavioral, and/or mood in offspring, increasing the risk of depression during adolescence and adulthood, and linked to perinatal suicides and maternal mortality in the first 12 months after delivery [11,12,13]. The COVID-19 pandemic changed people’s health behavior and sparked a psychological response reaction in society [14], especially in the pregnant population, currently suffering from an overall increased severity of anxiety [15] and an increased incidence of anxiety and depression [16]. There has been a noted sensitivity to social risks, an increase in negative emotions linked to anxiety and depression, indignation, and a decrease in positive emotions and life satisfaction after the declaration of COVID-19 in China related to the general human population [17].

The aim of this study was to analyze adverse mental health effects in the pregnant population during the COVID-19 pandemic, investigate risk factors for adverse mental health outcomes, identify protective factors, and create practical implications of mental health prophylaxis for clinical practice, bearing in mind the need to increase efforts in perinatal mental healthcare during such a pandemic.

## 2. Materials and Methods

A systematic scoping review [18] was conducted in electronic databases PubMed and Web of Sciences for keywords COVID-19, pregnancy, depression, anxiety, telemedicine for relevant critical articles (*n* = 3280) published from 2020 until October 2021 outlining the outcomes of control studies, meta-analysis, cross-sectional studies, face-to-face evaluation survey studies, remotely administered survey studies, and observational studies regarding the main topic, which were then evaluated. The equator checklist document used in this systematic scoping review was PRISMA Extension for Scoping Reviews [19] to analyze the presence of adverse mental health symptoms in pregnant women linked to the COVID-19 pandemic in domains as follows: social and medical consequences of COVID-19, psychological factors responsible for adverse COVID-19 mental health outcomes in pregnant women, the determination of COVID-19 mental health problems, including symptoms and diagnosis among pregnant women in different countries of the world, the influence of COVID-19 on psychological and medical factors related to adverse pregnancy and offspring development outcomes, and dilemmas and hopes in ways to improve the provision of services to pregnant women during disasters, including the COVID-19 pandemic.

### 2.1. Literature Search and Study Selection

A search was conducted on the PubMed and Web of Science databases. The search strategy included keywords linked to coronavirus, psychological symptoms, depression, and pregnancy. MESH terms (e.g., “pregnancy” (Mesh) AND “coronavirus” (Mesh) AND (“depression” (Mesh) OR “depressive disorder” (Mesh) OR “anxiety” (Mesh) OR “telemedicine” (Mesh) and text word search terms (“pregnancy” AND “coronavirus” AND (“mental health” OR “depression” OR “anxiety” OR “telemedicine”) were used.

### 2.2. Inclusion and Exclusion Criteria

The inclusion criteria were as follows: (1) any study including outcomes of control studies, meta-analysis, cross-sectional studies, face-to-face evaluation survey studies, remotely administered survey studies, and observational studies that recruited pregnant women with adverse mental health outcomes that resulted from the pregnant women’s experience with, exposure to, or infection with COVID-19; (2) studies written in English; (3) articles published from 2020 until October 2021; and (4) any original paper appearing in a peer-reviewed journal.

### 2.3. Data Collection

**Figure 1:**
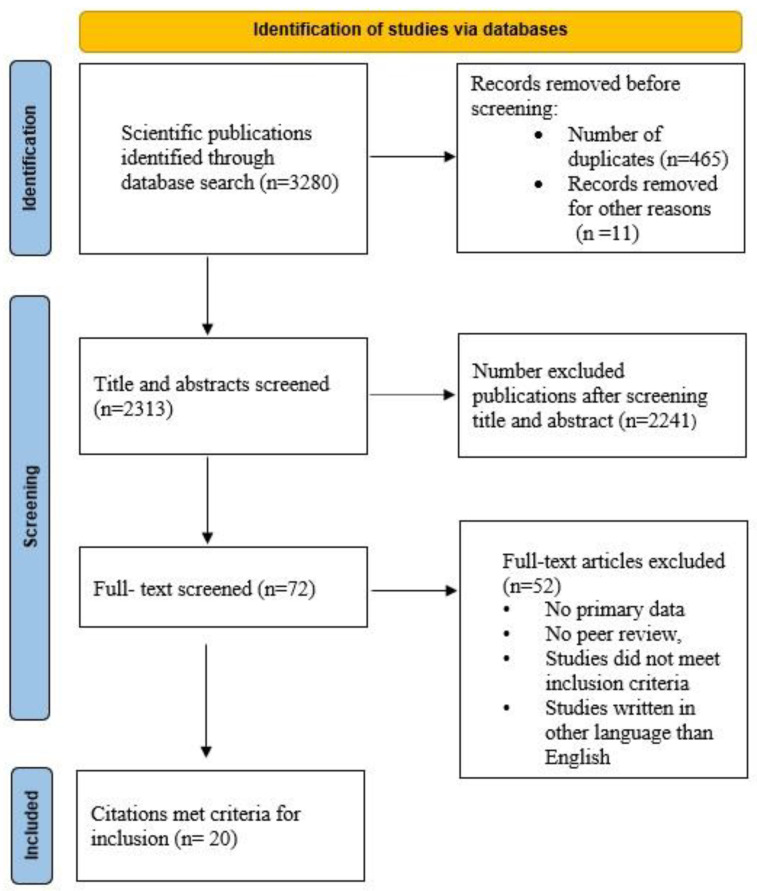
PRISMA flow diagram of the screening process.

Two researchers used a multistep approach to select eligible studies. In total, 3280 publications were retrieved in our study; duplicates, publications, upon title and abstract review, not meeting the research criteria, publications not linked to the research topic, or those not meeting the inclusion criteria after full-text evaluation were removed. In the case of disagreement among researchers, it was resolved during a consensus session with a third researcher. After a full-text review, 16 articles met the inclusion criteria. Figure 1 represents the PRISMA flow diagram summarizing the screening process.

### 2.4. Data Extraction and Investigated Variables

Two researchers independently extracted the following data: the aim of the study, country of the study, sample size, study design and assessment tool, and summary of study benefits and limitations. The publications strictly focused on the adverse mental health outcomes of the COVID-19 pandemic in pregnant women population, and research topic publications that met the criteria for inclusion were linked. Data were processed independently by two researchers and are summarized in Table 1.

### 2.5. Quality Assessment Tool

The qualitative Newcastle–Ottawa Quality Assessment Scale (NOQAS) [36] and the adapted version for cross-sectional studies to score each study (Supplementary Materials) were used. This tool on the basis of the criteria included 3 categories (“selection”, “comparability”, and “outcome”), with a maximal score of 9 and 10 points for cohort and cross-sectional studies, respectively. The “selection” category, which accounted for a maximum of 4 points (5 points for cross-sectional studies), the “comparability” category, which accounted for a maximum of 2 points, and “outcome,” which accounted for a maximum of 3 points. The quality of each study is shown in Table 1.

## 3. Results

### 3.1. Characteristics of Included Studies

**Figure 2:**
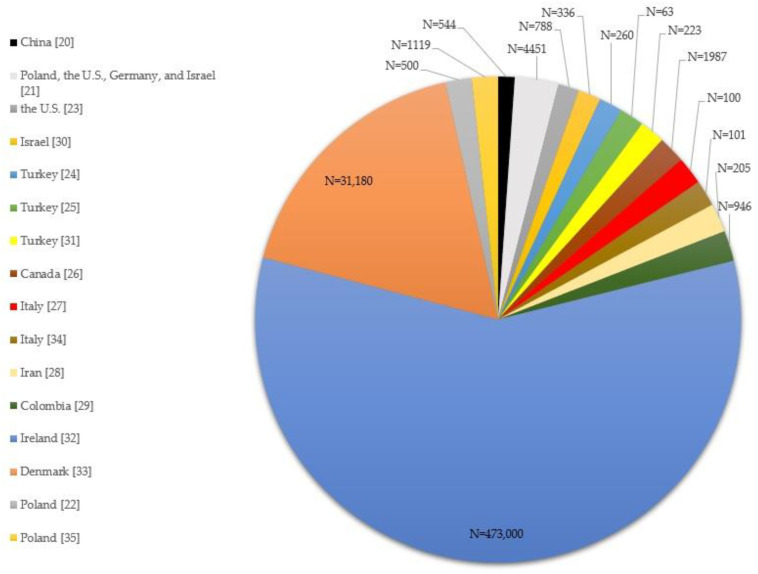
A total number of 515, 803 pregnant participants by the included sixteen studies.

Qualitative synthesis findings were concluded on the basis of 16 articles out of 3280 researched publications. A total of sixteen studies included in the final analysis: one study was conducted in China, Canada, Iran, Colombia, Ireland, Denmark, and Israel; two studies were done in the U.S., Poland, and Italy; three studies were conducted in Turkey. These publications described research into adverse mental health outcomes that resulted from a total number of 515,803 pregnant women’s experience with, exposure to, infection with COVID-19 in the following studies (Figure 2): China (N = 544) [20]; Poland, the U.S., Germany, and Israel (N = 4451) [21]; the U.S. (N = 788) [23]; Israel (N = 336) [30]; Turkey (N = 260) [24], (N = 63) [25], and (N = 223) [31]; Canada (N = 1987) [26]; Italy (N = 100) [27] and (N = 101) [34]; Iran (N = 205) [28]; Colombia (N = 946) [29]; Ireland (N = 473,000) [32]; Denmark (N = 31,180) [33]; Poland (N = 500) [22] and (N = 1119) [35].

### 3.2. Quality of Included Studies

A qualitative analysis was conducted with necessary reciprocal translations for interpreting the evaluation score of 16 articles out of 3280 published studies, outlining the outcomes of control studies, meta-analysis, cross-sectional studies, face-to-face evaluation survey studies, remotely administered survey studies, and observational studies regarding the main topic, which were then evaluated. Overall, 75% (12/16) of the studies evaluated using the Newcastle–Ottawa scale had an overall low risk of bias. The risk of bias for medium in three of the 16 studies (18.75%), and high in one (6.25%). Our study analyzes the presence of adverse mental health symptoms in pregnant women linked to the COVID-19 pandemic, and allows for us to divide and describe findings in five domains as follows.

### 3.3. Domain 1: Social and Medical Consequences of COVID-19 in Pregnant Women Population

Pregnant women are worried about the COVID-19 pandemic and have difficulty in accessing professional medical help; feel insecure about exposure risk to the coronavirus when accessing medical facilities; infection of the infant in the peripartum period; financial problems lead to conflict in the family; a single mother may be more prone to anxiety isolation, fear of being trapped and rumors spreading on social media, growing anxiety and social panic; and fear of blame, guilt and stigmatization related to being infected with COVID-19 [22]. A fear of infection, anger, and confusion related to financial status, ennui, inadequate information and supplies, and quarantine duration increased post-traumatic stress disorder (PTSD) symptoms and feelings of vulnerability [26,37,38,39,40,41], and all undeniably badly affect the emotional and mental statuses of pregnant women. Pregnant women are more likely to develop mental health problems than nonpregnant women of childbearing age are, and this was confirmed on the basis of a cross-sectional study of pregnant (*n* = 544) and nonpregnant (*n* = 315) women in Beijing, China [20]. The findings about the suppression of immune system function in pregnant women allow for us to classify the population of pregnant women into a group at high risk of SARS-CoV-2 infection [42]; on other hand, this population does not appear to be at increased risk of contracting SARS-CoV-2, since there is scientific confirmation that their rates of infection seem to parallel the rates of infection in their surrounding communities [43]. Women are more vulnerable and arguably less resistant to viral infection during the SARS-CoV-2 pandemic, as women and younger convalescent COVID-19 plasma donors are more likely to lack measurable neutralizing antibodies, while higher antibody levels were observed in men, older donors, and those who had been hospitalized [44]. Maternal risk factors associated with severe COVID-19 and admission to an intensive care unit are increasing age, high body mass, any preexisting maternal comorbidity, chronic hypertension, pre-eclampsia, preexisting diabetes, and both non-White ethnicity and high body mass index, are maternal risk factors for death and the need for invasive ventilation [8]. Unfortunately, evidence now exists for the maternal–fetal transmission of SARS-CoV-2, and N proteins were strongly expressed in the placenta of a COVID-19-positive pregnant woman whose newborn tested positive for viral RNA and developed COVID-19 pneumonia soon after birth [34]. The intrauterine transmission of SARS-CoV-2 appears to be rare, and this is possibly related to low levels of SARS-CoV-2 viremia and a decreased co-expression of ACE2 and TMPRSS2 needed for SARS-CoV-2 entry into the cells in the placenta [45]. The mode and timing of delivery should be individualized based on the severity of disease, existing comorbidities, and obstetric indications [46]. However, early cord clamping may minimize the risk of viral transmission by avoiding longer, close contact with the infected mother [46], and in turn, it may increase the mother’s good feelings about the safety of the newborn.

### 3.4. Domain 2: Main Psychological Factors Responsible for Adverse COVID-19 Mental Health Outcomes in Pregnant Women

Scientific publications reported that it is unclear whether and how SARS-CoV-2 can be transmitted from the mother to the fetus [47] and whether vertical transmission or placental pathology might occur following maternal infection during pregnancy remains unknown [48]. Women experience elevated levels of stress related to being worried about perinatal infection [35]. Fears of perinatal COVID-19 infection (29.1% of 4451 pregnant women in the U.S.) and feeling unprepared for birth due to the COVID-19 pandemic (nearly 30% of 4451 pregnant women in the U.S.) are noted as two major pandemic-related stressor domains for pregnant women in Poland, the U.S., Germany, and Israel [13]. We are faced with a paradox, because quarantine in the COVID-19 pandemic exacerbates the stress of isolation in pregnant women when a social connection helps us to cope with stress and maintain resilience [49].

### 3.5. Domain 3: Determination of COVID-19 Mental Health Problems Including Symptoms and Diagnosis among Pregnant Women in Different Countries

Even though 3–5% of the general population is affected by anxiety symptoms, a severe stressor such as the COVID-19 pandemic can increase such a rate in the general population of pregnant women if the prevalence of gestational anxiety is between 15% and 23%, as confirmed by scientific data [22]. COVID-19 pandemic-related stress predicts heightened anxiety in pregnant women during this crisis [23]. A preliminary study in Turkey on the effects of the COVID-19 pandemic on anxiety and depressive symptoms in pregnant women confirmed such a fact in 35.4% of the population; the obtained scores were higher than 13 on the Edinburgh Postpartum Depression Scale (EPDS) among respondents (*n* = 260) [24]; a score of more than 10 on the EPDS suggests that minor or major depression may be present [50]. The level of anxiety and depressive symptoms in pregnant women during COVID-19 infection significantly increased before such an event, and the pandemic outbreak leads to adverse birth outcomes [25]. However, a Canadian study indicated that pregnant women did not have higher levels of depression, stress, and anxiety prior the COVID-19 outbreak when compared with the time after the outbreak outcomes [51]. A study on elevated depressive and anxiety symptoms among pregnant individuals during the COVID-19 pandemic in Canada were surveyed, and its results provided information about substantially elevated anxiety and depressive symptoms compared to those in similar pre-pandemic pregnancy cohorts, with 37% reporting clinically relevant symptoms of depression and 57% reporting clinically relevant symptoms of anxiety; it also indicated stressor concerns about not receiving the necessary prenatal care, having relationship strain and social isolation due to the COVID-19 pandemic; more physical activity was associated with lower psychological symptoms [26]. A study conducted in Naples, Italy highlighted that participants who had never had depression or anxiety in previous pregnancy rated the psychological impact of the COVID-19 outbreak as severe (over 50%), especially in the first trimester of pregnancy during the outbreak, reported higher than normal anxiety in general (66%), and high anxiety regarding the vertical transmission of the disease was reported by almost half of the respondents [27].

An online descriptive–analytical cross-sectional study during the outbreak of COVID-19 with the DASS-21 tool (Depression, Anxiety and Stress Scale 21, which includes 21 questions and three subscales of stress, depression, and anxiety, and contains seven questions for each subscale, scored from not at all (0) to very high (3) for each question with 205 pregnant women in Iran, produced mean (SD) scores of depression, stress, and anxiety of 3.91 (3.9), 6.22 (4.25), and 3.79 (3.39); where depression, stress, and anxiety symptoms were observed in 32.7%, 32.7%, and 43.9% of the participants, from a mild to very severe degree [28]. In 2020, the clinical impact, psychological effects, and knowledge of pregnant women during the COVID-19 outbreak in seven cities in Colombia were evaluated, where 49.1% of women reported suffering from insomnia [29]. In comparison with the Turkish study [24], findings show that a lower rate of depressive symptoms (25%) was reported in Colombian work [29], while the same Colombian study confirmed a similar anxiety symptom rate (50.4%) with findings by Italian scientists [27], and higher than that in Iran’s study results [28]. A study on distress and anxiety among Jewish and Arab pregnant women in Israel in three domains on levels of all COVID-19-related anxieties were quite high, especially in Arab women, with concern over the health of the fetus, public transportation and place, being infected themselves, and the delivery of the baby [30]. In face-to-face evaluation surveys, EPDS and the Maternal Attachment Inventory were adopted in a study (conducted in COVID-19 pandemic referral hospital in Ankara, Turkey) and applied within 48 h after birth of the patients regarding the effect of the COVID-19 pandemic and social restrictions on depression rates and maternal attachment in immediate postpartum women; the study result highlighted that the rate of depression was twice lower in comparison with the reported findings of a preliminary study on depression and anxiety symptoms in pregnant Turkish women (35.4% rate of women had EPDS scores higher than 13) by Durankus and Aksu [24]; depressive symptoms of new mothers may have been positively impacted by appropriate isolation in hospitals for pregnant women, so the feeling of being safe and isolated may be the cause of better EPDS scores [31].

### 3.6. Domain 4: Influence of COVID-19 on Psychological and Medical Factors Related to Adverse Pregnancy and Offspring Development Outcomes

Influence of COVID-19 on medical factors may be linked to adverse pregnancy and offspring development outcomes. Hyperinflammation as a symptom of SARS-CoV-2 reported in pregnant women suffering from SARS-CoV-2 infection, and cytokine storms could hypothetically adversely increase the risk for neurodevelopmental disorders in the neonates [3]. In cases of acute respiratory syndrome coronavirus (SARS-CoV) and MERS-CoV, high incidences of spontaneous miscarriage, preterm birth, and neonatal death have been reported [52,53]. However, a study was performed in China on the effects of SARS-CoV-2 infection during pregnancy (*n* = 35) versus nonpregnant patients, and its findings on the clinical symptoms of COVID-19 in pregnant women were misleading and atypical (compared with those in 31 nonpregnant patients), with a significantly lower proportion of fever (54.8% vs. 87.5%, *p* = 0.006), a shorter average interval from onset to hospitalization, and a higher proportion of severe or critical COVID-19 (32.3% vs. 11.4%, *p* = 0.039) [54].

The influence of COVID-19 on psychological factors may be related to adverse pregnancy and offspring development outcomes, too. There is a clear link between poor mental health in pregnant women and pregnancy complications [10]. The exposure of pregnant women to psychological distress, even linked with depression and stress in general or anxiety, is a risk factor of schizophrenia spectrum disorders, autism spectrum disorder (ASD), antisocial behavior, and attention deficit hyperactivity disorder (ADHD) [55,56]; thus, the psychological stress of the COVID-19 pandemic during pregnancy can increase the risk of neurodevelopmental disorders in offspring [57], and the risk preterm delivery, low birth weight, and postnatal complications [58]. The COVID-19 lockdown may have adverse birth weight outcomes. An unprecedented reduction in births of very low birth weights (VLBW) and extremely low birth weights (ELBW) infants was reported in the general population during the COVID-19 lockdown in Ireland [32] and Denmark [33].

### 3.7. Domain 5: Dilemmas and Hopes in Ways to Improve the Provision of Services to Pregnant Women during Disasters, Including the COVID-19 Pandemic

Providing support and care in psychopsychiatric services for pregnant women is a priority. It may be difficult during the COVID-19 pandemic if a mental health provider system is lacking and healthcare professionals are not trained in the field of mental health, as was confirmed in the case of Kashmir, India [59]. In survey research conducted in Belgium, only 38.2% of women received, and 61.8% of respondents did not receive, medical help from their obstetrician because of the COVID-19 pandemic [60]. The scope of psychological and psychiatric services for pregnant women is also changing in favor of telemedicine [23] to have remote access to a psychiatrist and provide psychological therapy for pregnant women and even continue such service after the delivery of a baby. This option is recommended, especially during the COVID-19 pandemic, by the American College of Obstetricians and Gynecologists (ACOG) and the Society for Maternal and Fetal Medicine (SMFM) [61,62]. Research conducted in Poland demonstrated that 47.41% of women had at least one telehealth appointment during their pregnancy, which is a much higher percentage rate than 31.8% of telehealth appointments in New York during the COVID-19 pandemic [63]. The same study concluded that it is preferable to recommend a hybrid healthcare model over a traditional in-person care model, and patient testing and screening should be performed in person, while follow-up visits can be carried out via telehealth, and the recommended model allows for providers to lower the risk of COVID-19 infection while maintaining a high standard of prenatal care [63]. The education of midwives is a high priority to address the effects of a life-threatening mass disaster event, the health of a pregnant woman and her fetus, the course of her pregnancy and delivery, the methods of prevention and treatment, and extending professional authorizations under the midwife license in the face of terrorism and/or mass disaster [64].

## 4. Discussion

The COVID-19 pandemic formed a serious multi-etiological global mental health challenge influencing every aspect of life and disrupting the social fabric [65]. The COVID-19 pandemic has caused general anxiety worldwide [66] and created numerous stressful conditions, especially for vulnerable populations such as pregnant women [35]; in some cases, the increased the risk of psychological imbalance associated with changes in everyday life [67] and increased the risk of the most common mental disorders among pregnant women during this a worldwide disaster [68], including anxiety, depression, insomnia, and PTSD [20,22,23,24,29]. The aim of this study was to analyze adverse mental health effects in the pregnant population during the COVID-19 pandemic, investigate risk factors for adverse mental health outcomes, identify protective factors, and create practical implications for clinical practice, bearing in mind the need to improve perinatal mental healthcare during such pandemics. Qualitative research was conducted in the electronic databases PubMed and Web of Sciences for the keywords COVID-19, pregnancy, depression, anxiety, and telemedicine for relevant critical articles (*n* = 3280) published from 2020 until October 2021, outlining the outcomes of control studies, meta-analysis, cross-sectional studies, face-to-face evaluation survey studies, remotely administered survey studies, and observational studies regarding the main topic; all were evaluated. The qualitative synthesis findings were concluded on the basis of 16 articles out of 3280 researched publications. Our findings on a total number of 515,803 pregnant participants are linked to adverse mental health sequelae of the COVID-19 pandemic in the pregnant population. Our study analyzes the presence of adverse mental health symptoms in pregnant women associated with the COVID-19 pandemic and allows us to divide and describe findings in five domains: Domain 1: Social and medical consequences of COVID-19 in the pregnant women population. Domain 2: Main psychological factors responsible for adverse COVID-19 mental health outcomes in pregnant women. Domain 3: Determination of COVID-19 mental health problems, including symptoms and diagnosis among pregnant women in different countries. Domain 4: Influence of COVID-19 on the psychological and medical factors related to adverse pregnancy and offspring development outcomes. Domain 5: Dilemmas and hopes in ways to improve the provision of services to pregnant women during disasters, including the COVID-19 pandemic. Pregnant women are more likely to develop mental health problems than nonpregnant women of childbearing age are, and this was confirmed on the basis of a cross-sectional study of pregnant (*n* = 544) and nonpregnant (*n* = 315) women in Beijing, China [20]. Elevated anxiety and depression symptoms that may have a long-term impact on offspring related to COVID-19 worries about threats to their own lives and their baby’s health [23], not getting enough prenatal care, and social isolation were the most frequently reported factors in the population of 1987 women <35 weeks gestation [26]. Evidence from 38.5% of participants reported high preparedness stress; 26% reported high perinatal infection stress, and pregnant women are most vulnerable to pandemic-related stress [35]. Pregnant women have an advantage of facing mental problems related to COVID-19, showing fewer depression, anxiety, insomnia, and PTSD symptoms than nonpregnant women do [20]. However, the effects of the COVID-19 pandemic on mental health are still not fully understood; thus, research in this area is still being conducted in many countries—among others, in Turkey, Colombia, and Italy. In comparison with the Turkish study [24], findings show that a lower rate of depressive symptoms (25%) was reported in Colombia [29], while the same Colombian study confirmed similar anxiety symptom rate (50.4%) with findings pointed out by Italian scientists [27] and higher than that in Iran’s study results [28]. We noticed two major pandemic-related stress domains for pregnant women: fear of perinatal COVID-19 infection and being unprepared for birth were reported in four countries [21]. Pregnant women are worried about the COVID-19 pandemic and have difficulty in accessing professional medical help, feel insecure about the exposure risk to the coronavirus when accessing medical facilities, fear infection of the infant in the peripartum period, and have financial problems that lead to conflict in the family; additionally, a single mother may be more prone to anxiety isolation; fear of being trapped and rumors spreading on social media; growing anxiety and social panic; and fear of blame, guilt, and stigmatization related to being infected with COVID-19 [22]. Psychological impact and anxiety of the COVID-19 epidemic were found be more severe in women who were in the first trimester of pregnancy during the outbreak; high anxiety regarding the vertical transmission of the disease was reported by almost half of the respondents [27]. Notable, the rate of psychological consequences of the pandemic was much larger than the number of patients clinically affected by the virus, with symptoms of anxiety, insomnia, and depression [29]. Depressive and anxiety symptoms were significantly increased during the SARS-CoV-2 pandemic compared with pre-pandemic surveys, and effective screening strategies for depression and anxiety symptoms during the pandemic should be prioritized to allow for timely treatment [25]. Lockdowns (e.g., reduced infection load and reduced physical activity) are possibly beneficial for reducing extreme prematurity and potentially reducing infant mortality, a nonsignificant but slightly increased number of very premature births [33]. Evidence from pregnant women in China found that low levels of physical activity were associated with increased symptoms of depression during COVID-19, and in Canada, pregnant women who engaged in higher levels of physical activity tended to show lower symptoms of depression and anxiety [69]. The pregnant woman population is struggling with the COVID-19 pandemic, experiencing stressful factors such as adverse fetal outcomes and the increase in ruptured ectopic pregnancies, causing maternal deaths and stillbirths, concerns about the spread or infection of SARS-CoV-2, financial instability, limited scientific knowledge about the effects of COVID-19 on fetal wellbeing, and interruptions of prenatal care [35]. Urgent need to provide psychosocial support to the pregnant women population during the COVID-19 crisis was highlighted. Adverse events may otherwise occur during pregnancy and thus affect both mother and fetus [24].

Our study produced five overarching domains by analysis and synthesis of the current knowledge on the main topic related to adverse consequences of the COVID-19 on the mental health of pregnant women and the possibility of improving these outcomes in a medical provider practice in a way to reach the preparation of useful implications for clinical practice during the COVID-19 pandemic.

### 4.1. Useful Implications for Clinical Practice

Research findings support and advocate the need to modify the scope of healthcare provider practice in the COVID-19 pandemic disaster and could be implemented and adopted by healthcare providers, as useful implications for clinical practice on the following discoveries:

- psychopsychiatric consequences of the negative influence of COVID-19 globally affect women regardless of race of origin [10,20,21,23,24,25,26,27,28,29,30,31,32,33,35,51,52,53,54,57,59,70];
- fears of perinatal COVID-19 infection and feeling unprepared for birth due to the COVID-19 pandemic can be a major psychological problem for pregnant women [21,27];
- quarantine may involve destructive feelings of loss of freedom among pregnant women [49], and social distancing and isolation/quarantine procedures implemented during the COVID-19 pandemic increased risk of psychological problems among pregnant women and new mothers [71];
- pregnant women should be informed of the increase in severity of COVID-19, including admission to intensive care units, need for ECMO and invasive ventilation compared with non-pregnant women, and encouraged to undertake safety measures to reduce the risk of infection, and pregnant women with preexisting comorbidities will need to be considered as a high-risk group for COVID-19 [8];
- the possibility of a mother infecting her own unborn child with the SARS-CoV-2 virus is an extreme psychological burden for the mother and a serious factor that may impair the child’s development [27,35];
- early cord clamping may minimize the risk of viral transmission by avoiding longer, close contact with the infected mother [46], and in turn, it may increase the mother’s good feelings about the safety of the newborn;
- mental health problems among pregnant women linked to the COVID-19 pandemic in most cases establish symptoms of depression, anxiety, insomnia, and PTSD [20,22,23,24,29];
- the status of not receiving the necessary prenatal care, having relationship strain, and being in social isolation may become initiating factors of mental health disorders [35,59];
- a positive impact on the depressive symptoms of new mothers may be achieved by providing appropriate isolation in hospitals for pregnant women [31], including. but not limited to. quarantine;
- the psychological stress of the COVID-19 pandemic during pregnancy can increase the risk of preterm delivery, low birth weight, postnatal complications, and neurodevelopmental disorders in offspring [10,25,57,58]; in some cases, a 100% reduction in ELBW infants was noted during the COVID-19 pandemic [32,33];
- telemedicine may be used to effectively improve access to medical services for women during the pandemic, especially in quarantined areas, reducing feelings of fear, threat, and loneliness [23,61,72], and may improve access and utilization of prenatal care across the board [63,70];
- reducing feelings of fear, panic, anxiety, threat, and loneliness is a priority, especially during a disaster [23,61,72,73];
- a midwife should have the right and duty to order, prescribe, and administer pharmacological agents that, on a daily basis, are prescribed at the discretion of an OB/GYN specialist and must be prepared for sudden maternal cardiac arrest in a pregnant woman and to address the moral dilemma of delivering a fetus from a deceased mother’s womb [64];
- critically important are methods of supportive parenting and using techniques to downregulate arousal in time of occurred stress, and physical activity was associated with lower psychological symptoms [26];
- effective screening strategies for depressive and anxiety symptoms during the pandemic should be prioritized to allow for timely treatment [25];
- mental health specialists and other medical providers, midwives, and nurses can prevent adverse outcomes by identifying problems early (paying special attention to the group of women with adverse mental health and psychiatric symptoms, including assessing sleep patterns, sources of fear, anxiety, swinging moods, irritability, depression, worries, and suicidal ideation or its attempt), and establishing comprehensive treatment plans for pregnant women in conditions such as emergencies and the COVID-19 natural disasters [25];
- routine assessment of trauma history and psychopathology during prenatal visits is warranted to identify women at risk; abnormalities of the early bonding of mother–offspring always must be taken into consideration, if mental health impairment symptoms occur [74], and in the case of necessity, a perinatal psychiatrist should be consulted [75];
- promoting marital and relationship wellbeing may play a valuable role in anxiety control, lowering stress, and reducing depression in pregnant women [28];
- physical exercise is often correlated with decreased depressive and anxiety symptoms in pregnant women and therefore should be recommended [69,76];
- patient testing and screening should be conducted in person, while follow-up visits can be carried out via telehealth, and the recommended model allows for providers to lower the risk COVID-19 infection while maintaining a high standard of prenatal care [63];
- women who became mothers during the COVID-19 emergency appear to be at high risk for developing mental health problems [5];
- implementing community-based strategies to support resilience and psychologically vulnerable individuals during the COVID-19 crisis is fundamental for any community [77], including pregnant women;
- copying with psychological distress of the COVID-19 pandemic during pregnancy should be more recommended to prevent adverse effects on the fetal growth and neurodevelopment disorders in offspring, because maternal psychological distress (e.g., stress, anxiety, and depression) has been found as a risk factor of child or adult neurodevelopment disorders, including, but not limited to, ADHD, ASD, schizophrenia spectrum disorder, antisocial behavior, and depressive symptoms [3,55,56,57];
- observation highlights the need for increased screening and treatment for perinatal mood and anxiety disorders in the postpartum period as the COVID-19 pandemic continues [75];
- providing psychological support to pregnant and lactating women may reduce the long-term negative effects of COVID-19 pandemic [76].

### 4.2. Strengths and Limitations

The strength of this study is that it was conducted with the use of and following up the PRISMA extension for scoping review guidelines. The limitations of this study may have resulted from the inclusion criterion of researching articles only written in English language, which could have excluded valuable scientific publications in other languages.

## 5. Conclusions

Our study analyzed the presence of adverse mental health symptoms in a total number of 515,803 pregnant women’s experience with exposure to or infection with COVID-19. The psychological impact and anxiety of the COVID-19 crisis were found be more severe in women who were in the first trimester of pregnancy during the disaster. Depression symptoms worries about threats to their own lives, their baby’s health, not getting enough prenatal care, and social isolation are some of the most important factors in group of <35 weeks gestation age; 26% pregnant women reported perinatal COVID-19 infection stress and being unprepared for birth. Depressive and anxiety symptoms were significantly increased during the SARS-CoV-2 pandemic compared with pre-pandemic surveys.

Urgent need to provide psychosocial support to this population during the crisis was highlighted and allowed us to produce the useful implications for clinical practice bearing in mind adverse mental health effects in the pregnant population during the COVID-19 pandemic and following our efforts to investigate risk factors for adverse mental health outcomes, identify protective factors and the need to improve perinatal mental healthcare during such pandemics. The useful implications for clinical practice to improve the adverse mental health outcomes of pregnant women associated with the COVID-19 pandemic are highly desirable. This is necessary to not only eliminate the long-term consequences of different forms of severe stressors’ outcomes but also to reduce the economic costs of medically treating adverse severe mental problems. Improving the effectiveness of midwifery and nursing practice for such disaster responses is pivotal. To address adverse mental health issues during the pandemic, it is necessary to adopt several psychosocial interventions to reduce anxiety and other destructive mental health outcomes among the population of pregnant women with methods for controlling outbreaks like COVID-19. It is urgent to conduct cohort studies of the impact of the COVID-19 pandemic on the mental state of pregnant women, fetuses, and child development outside the mother’s womb.

## Figures and Tables

**Table 1:** Qualitative synthesis findings.

Aim of Study	Country	Sample Size	Study Design and Assessment Tool	Study Summary Benefits	Limitations	Study Quality
Prevalence of psychiatric symptoms of pregnant and non-pregnant women during the COVID-19 epidemic (Zhou et al., 2020) [20]	China	N = 544 pregnant; *n* = 315 nonpregnant.	Cross-sectional study: used social media application; online patient health questionnaire (PHQ-9); generalized anxiety disorder scale (GAD-7); somatization subscale of the symptom checklist 90 (SCL-90); insomnia severity index (ISI); post-traumatic stress disorder checklist-5 (PCL-5).	Pregnant women have an advantage of facing mental problems caused by COVID-19, showing fewer depression, anxiety, insomnia, and PTSD symptoms than nonpregnant women do.	Lacks longitudinal follow-up, limiting the generalization of findings to other regions; No data on psychological interventions for pregnant women.	8/10
Vulnerability and resilience to pandemic-related stress among U.S. woman pregnant at the start of the COVID-19 pandemic (Preis et al., 2020.a.) [21]	U.S.	N = 4451	Cross-sectional study: secure online software survey Questionnaire. The Pandemic-Related Pregnancy Stress Scale (PREPS).	Two major pandemic-related stress domains for pregnant women in Poland, the U.S., Germany, Israel: fears of perinatal COVID-19 infection, and being unprepared for birth.	Excluded women without access to the Internet and social media.	7/10
Risk factors for anxiety and depression among pregnant women during the COVID-19 pandemic: web-based cross-sectional survey (Kajdy et al., 2020) [22]	Poland	N = 500	Web-based cross-sectional survey: GAD-7; PHQ-9. Available in 15 languages.	Pregnant women are worried about the COVID-19 pandemic and have difficulty in accessing professional medical help; feel insecure about exposure risk to the coronavirus when accessing medical facilities; infection of the infant in the peripartum period; financial problems lead to conflict in the family; a single mother may be more prone to anxiety isolation, fear of being trapped and rumors spreading on social media, growing anxiety and social panic; fear of blame, guilt and stigmatization related to being infected with COVID-19.	Survey may reach more women of a higher socioeconomic status and from larger agglomerations.	9/10
Pandemic-related pregnancy stress and anxiety among women pregnant during the coronavirus disease 2019 pandemic. (Preis et al., 2020.b.) [23]	U.S.	N = 788	Cross-sectional study: social media to complete online questionnaire GAD-7; PREPS.	COVID-19 pandemic-related stress predicts heightened anxiety in women pregnant during this crisis: preparation for birth; worries about COVID-19 infection to self and baby.	Inclusion criteria: pregnant at the time of questionnaire completion and older than 18 years; exclusion was inability to read or write English.	8/10
Effects of the COVID-19 pandemic on anxiety and depressive symptoms in pregnant women: preliminary study. (Durankuş and Aksu, 2020) [24]	Turkey	N = 260 out of 318	Cross-sectional study: online questionnaire survey study EPDS-Edinburgh Postpartum Depression Scale.	Urgent need to provide psychosocial support to this population during the crisis. Adverse events may otherwise occur during pregnancy, and thus affect both mother and fetus.	Survey was administered online, thus preventing a face-to-face evaluation of participants; authors used their own created questionnaire on the pandemic, and its psychological effects were subjective.	6/10
Anxiety and depression symptoms in the same pregnant women before and during the COVID-19 pandemic (Ayaz et al., 2020) [25]	Turkey	N = 63	Cross-sectional study: Beck Anxiety Inventory (BAI) questionnaire; Depression and Anxiety Symptoms II (IDAS II) questionnaire.	Depressive and anxiety symptoms were significantly increased during the SARS-CoV-2 pandemic compared with pre-pandemic surveys. Effective screening strategies for depression and anxiety symptoms during the pandemic should be prioritized to allow for timely treatment.	Sample size, but power analysis indicated that the effect of this limitation was reduced.	3/10
Elevated depression and anxiety symptoms among pregnant individuals during the COVID-19 pandemic (Lebel et al., 2020) [26]	Canada	N = 1987 <35 weeks gestation	Cross-sectional study: Online survey of standardized measures of depression, anxiety, pregnancy-related anxiety, and social support. EPDS; PROMIS Anxiety Adult 7-item short form; social support effectiveness questionnaire (SSEQ); interpersonal support evaluation list (ISEL); Godin-Shephard Leisure-Time Exercise Questionnaire.	Elevated anxiety and depression symptoms that may have a long-term impact on offspring related to COVID-19 worries about threats to their own lives, their baby’s health, not getting enough prenatal care, and social isolation.	Inclusion criteria: living in Canada, able to read and write English, and having a confirmed pregnancy <35 weeks gestation.	9/10
Psychological impact of coronavirus disease 2019 in pregnant women (Saccone et al., 2020) [27]	Italy	N = 100	Cross-sectional study: Event Scale-Revised (IES-R) questionnaire; Spielberger State-Trait Anxiety Inventory (STAI); visual analog scale (VAS).	Psychological impact and anxiety of the COVID-19 epidemic found be more severe in women who are in the first trimester of pregnancy during the outbreak; high anxiety regarding the vertical transmission of the disease was reported by almost half of the respondents.	Findings from the study were limited by the single-center study design and small sample size.	5/10
Depression, stress, anxiety, and their predictors in Iranian pregnant women during the outbreak of COVID-19 (Effati-Daryani et al., 2020) [28]	Iran	N = 205	Online descriptive–analytical cross-sectional study; Depression, Anxiety and Stress Scale 21 (DASS-21).	Promoting marital life satisfaction and socioeconomic status can play an effective role in controlling anxiety, and reducing stress and depression in pregnant women.	Those who had a mobile phone with Internet connection could participate in this study.	7/10
Attitudes and collateral psychological effects of COVID-19 in pregnant women in Colombia (Parra-Saavedra et al., 2020) [29]	Colombia	N = 946 out of 1021	Cross-sectional web survey.	Rate of psychological consequences of the pandemic was much larger than the number of patients clinically affected by the virus, with symptoms of anxiety, insomnia, and depression.	Excluded women without access to the Internet and social media.	8/10
Distress and anxiety associated with COVID-19 among Jewish and Arab pregnant women in Israel. (Taubman-Ben-Ari et al., 2020) [30]	Israel	N = 336 comprising 225 Jewish and 111 Arab pregnant women	Cross-sectional study: social media to complete online questionnaire. Mental Health Inventory- Short Form based on the original MHI.	COVID-19-related anxieties were quite high, especially in Arab women, with concern over the health of the fetus, public transportation and place, being infected themselves, and the delivery of the baby.	Cannot be considered representative of population of pregnant women in Israel, questionnaire only in Hebrew.	6/10
The effect of COVID-19 pandemic and social restrictions on depression rates and maternal attachment in immediate postpartum women: a preliminary study. (Oskovi-Kaplan et al., 2020) [31]	Turkey	N = 223	Cross-sectional study: EPDS and Maternal Attachment Inventory (MAI).	Positive impact on the depressive symptoms of new mothers may have providing appropriate isolation in hospitals; psychological status of pregnant and postpartum women may help in the improvement of psychosocial support.	Lack of a control group that was evaluated before the onset of pandemic and due to ongoing cases with a high incidence; a lack of any validated questionnaire for COVID-19 infection on psychological status.	7/10
Unprecedented reduction in births of very low birthweight (VLBW) and extremely low birthweight (ELBW) infants during the COVID-19 lockdown in Ireland: a ‘natural experiment’ allowing analysis of data from the prior two decades. (Philip et al., 2020) [32]	Ireland	N = 473,000	Descriptive cohort study: VON international benchmarking; labor ward weekly statistics for live and stillbirths; early pregnancy assessment unit (EPAU) statistics for early pregnancy loss/miscarriage information; inpatient ward statistics for early or late fetal loss during hospital admission.	100% reduction in ELBW infants was noted in one designated health region of Ireland from January to April 2020 compared with the preceding 20 years.	Retrospective nature of birth cohort data from one health region of Ireland; completion of the study prior to the official finish of lockdown; 3. ELBW cohort analyzed with the small number of births.	8/10
Danish premature birth rates during the COVID-19 lockdown. (Hedermann et al., 2020) [33]	Denmark	N = 31,180 live singleton infants	Cross-sectional study; Nationwide prevalence proportion study with premature births as cases, term pregnancies as controls, and birth during lockdown from 12 March to 14 April 2015–2020.	Lockdowns (e.g., reduced infection load and reduced physical activity) are possibly beneficial for reducing extreme prematurity and potentially reducing infant mortality; a nonsignificant but slightly increased number of very premature births.	Study summary benefits data need to be confirmed in other countries.	8/10
SARS-CoV-2 vertical transmission with adverse effects on the newborn revealed through integrated immunohistochemical, electron microscopy and molecular analyses of Placenta. (Facchetti et al., 2020) [34]	Italy	N = 101	Cross-sectional study; Research: comprehensive immunohistochemical and immune- fluorescence analysis: RNA-in situ hybridization and RT-PCR for S transcripts, and by electron microscopy.	First evidence for maternal–fetal transmission of SARS-CoV-2, likely propagated by circulating virus-infected fetal mononuclear cells.	No limitation reported.	9/10
Pandemic stress and its correlates among pregnant women during the second wave of COVID-19 in Poland (Ilska et al., 2021) [35]	Poland	N = 1119	Cross-sectional study design, online survey; PREPS.	38.5% of participants reported high preparedness stress; 26% reported high perinatal infection stress, pregnant women are most vulnerable to pandemic-related stress.	Excluded women who had no access to the Internet or social media.	8/10

## Supplementary Materials

The following supporting information can be downloaded at https://www.mdpi.com/article/10.3390/jcm11082072/s1: NEWCASTLE—OTTAWA QUALITY ASSESSMENT SCALE.
