# Supplementary material for: Adverse Mental Health Sequelae of COVID-19 Pandemic in the Pregnant Population and Useful Implications for Clinical Practice
Source: J Clin Med. 2022 Apr 7;11(8):2072. doi: 10.3390/jcm11082072 (PMC9032065; doi:10.3390/jcm11082072)
Supplement: Supplementary file 1 [file jcm-11-02072-s001.zip › jcm-1611468-supplementary.pdf]

Mazurkiewicz, D.W. et al. Adverse Mental Health Sequelae of COVID-19 Pandemic in the Pregnant Population and Useful Implications for Clinical Practice.

NEWCASTLE - OTTAWA QUALITY ASSESSMENT SCALE (adapted for cross sectional studies)

Selection: (Maximum 5 points)

1) Representativeness of the sample:

a) Truly representative of the average in the target population. (all subjects or random sampling)

b) Somewhat representative of the average in the target population. (nonrandom sampling)

c) Selected group of users.

d) No description of the sampling strategy.

2) Sample size:

a) Justified and satisfactory.

b) Not justified.

3) Non-respondents:

a) Comparability between respondents and non-respondents' characteristics is established, and the response rate is satisfactory.

b) The response rate is unsatisfactory, or the comparability between respondents and non-respondents is unsatisfactory.

c) No description of the response rate or the characteristics of the responders and the non-responders.

4) Ascertainment of the exposure (risk factor):

a) Validated measurement tool.

b) Non-validated measurement tool, but the tool is available or described.

c) No description of the measurement tool.

Comparability: (Maximum 2 points)

1) The subjects in different outcome groups are comparable, based on the study design or analysis. Confounding factors are controlled.

a) The study controls for the most important factor (select one).

b) The study control for any additional factor.

Outcome: (Maximum 3 points)

1) Assessment of the outcome:

a) Independent blind assessment.

b) Record linkage.

c) Self report.

d) No description.

2) Statistical test:

a) The statistical test used to analyze the data is clearly described and appropriate, and the measurement of the association is presented, including confidence intervals and the probability level (p value).

b) The statistical test is not appropriate, not described or incomplete.

This scale has been adapted from the Newcastle-Ottawa Quality Assessment Scale for cohort studies to perform a quality assessment of cross-sectional studies for the systematic review, “Adverse Mental Health Sequelae of COVID-19 Pandemic in the Pregnant Population and Useful Implications for Clinical Practice.”

In our scale, we have specifically assigned this tool on a basis of criteria included 3 categories (“selection”, “comparability”, “outcome”) with a maximum score of 9 and 10 points for cohort and cross-sectional studies respectively. The “selection” category, which accounts for a maximum of 4 points (5 points for cross-sectional studies), the “comparability” category, which accounts for a maximum of 2 points, and “outcome,” which accounts for a maximum of 3 points.
